# A non-inferiority study of the novel selective urate reabsorption inhibitor dotinurad versus febuxostat in hyperuricemic patients with or without gout

**DOI:** 10.1007/s10157-020-01851-6

**Published:** 2020-01-22

**Authors:** Tatsuo Hosoya, Kazuki Furuno, Shingo Kanda

**Affiliations:** 1 Jikei University School of Medicine, 3-25-8, Nishi-Shimbashi, Minato-ku, Tokyo 105-8461 Japan; 2 Clinical Research Department, Mochida Pharmaceutical Co., Ltd., 1-22 Yotsuya, Shinjuku-ku, Tokyo 160-0004 Japan

**Keywords:** Hyperuricemia, Gout, Selective urate reabsorption inhibitor (SURI), URAT1 inhibitor, Dotinurad, Febuxostat

## Abstract

**Background:**

Dotinurad is a novel, selective urate reabsorption inhibitor, which reduces serum uric acid levels by selective inhibition of the urate transporter 1. We evaluated the efficacy and safety of dotinurad versus febuxostat, a widely used drug in Japan, in hyperuricemic Japanese patients with or without gout.

**Methods:**

This was a multicenter, randomized, double-blind, active-controlled, parallel-group, forced-titration study in hyperuricemic patients. Study treatment in the dotinurad and febuxostat groups was initiated at 0.5 and 10 mg/day, followed by dose titration to 2 and 40 mg/day, respectively, over 14 weeks. The primary endpoint was the percent change in serum uric acid level from the baseline to the final visit.

**Results:**

A total of 203 hyperuricemic patients with or without gout were enrolled in the study and randomized to receive dotinurad or febuxostat. The percent change in serum uric acid level from the baseline to the final visit was 41.82% in the dotinurad group and 44.00% in the febuxostat group. The mean difference was − 2.17% (two-sided 95% confidence interval − 5.26% to 0.92%). The lower limit of the interval was above the non-inferiority margin (− 10%), demonstrating the non-inferiority of dotinurad to febuxostat. The profiles of adverse events and adverse drug reactions raised no noteworthy safety concerns in either group.

**Conclusion:**

The non-inferiority of dotinurad to febuxostat in terms of serum uric acid lowering effect was confirmed. No noteworthy safety concerns arose.

## Introduction

Hyperuricemia, defined in Japan as serum uric acid levels > 7.0 mg/dL regardless of sex or age, is a pathological condition that can cause urate deposition diseases including gouty arthritis [1, 2]. Appropriate control of uric acid levels may prevent uric acid deposition diseases, protect renal function in gouty patients who have renal impairment, and reduce cardiovascular risk [3, 4].

The Japanese guideline for the management of hyperuricemia and gout (second edition) states that hyperuricemia is caused by uric acid overproduction (overproduction type), uric acid underexcretion (underexcretion type), or their combination (combined type), accounting for approximately 10%, 60%, and 30%, respectively, of hyperuricemic patients in Japan. The guideline recommends the treatment of hyperuricemia using xanthine oxidoreductase inhibitors (XOIs) for the overproduction type and uricosuric drugs for the underexcretion type [5].

The recently revised guideline (third edition) classifies hyperuricemia into the following three types: the underexcretion type, the renal load type, or the combined type. The renal load type, a newly proposed type, is further divided into two subtypes: the overproduction type and the extrarenal underexcretion type. The extrarenal underexcretion type represents the state of decreased uric acid excretion from the intestinal tract due to hypofunction of ATP-binding cassette transporter G2 (ABCG2). It has become evident that this condition is likely to increase uric acid excretion from the kidneys [6], resulting in apparent overproduction of uric acid and making it impossible to distinguish the overproduction type from the extrarenal underexcretion type [2]. Therefore, patients classified as the overproduction type according to the conventional criteria who account for approximately 10% of the hyperuricemic population may include patients with impaired excretion.

On the basis of the current guideline (third edition), it can be assumed that patients with uric acid overproduction represent less than 10% of the hyperuricemic population and most patients have impaired uric acid excretion. However, uricosuric drugs are less prescribed and XOIs are widely used in clinical practice [7]. Febuxostat is being widely used these days owing to its advantages that dose reduction is not required in patients with renal dysfunction [8] and the drug is effective for all hyperuricemic types [9, 10]. On the other hand, the Food and Drug Administration has issued a warning that compared to allopurinol, febuxostat is associated with an increased risk of all-cause mortality including cardiovascular death according to the results of the CARES study [11]. However, the results in the FREED study were not consistent [12]. This warning is still controversial and warrants further investigation to ascertain whether febuxostat increases mortality risk.

Benzbromarone is the most prescribed uricosuric drug in Japan. Benzbromarone is reported to cause serious hepatic injury [13] and is contraindicated in patients with hepatic impairment in Japan. In addition, it has a potent inhibitory activity against cytochrome P450, with the suggestive potential for drug interactions [14]. Therefore, safe drugs with satisfactory efficacy need to be developed so that patients of the underexcretion type can receive treatment based on the mechanism of hyperuricemia.

Dotinurad, a novel selective urate reabsorption inhibitor (SURI) [15], reduces serum uric acid levels by selectively inhibiting urate transporter 1 (URAT1), which is expressed on the proximal renal tubules and is responsible for reabsorption of uric acid. In contrast, benzbromarone has inhibitory effects not only on URAT1, but also on organic anion transporter (OAT) 1 and OAT3, both of which are responsible in the kidneys for uric acid secretion in urine, and ATP-binding cassette transporter G2 (ABCG2), which is responsible for uric acid secretion from the intestinal tract and proximal tubule [15]. Both agents reduce serum uric acid levels through urinary uric acid excretion; however, the SURI dotinurad is expected to be more efficient than benzbromarone in reducing uric acid levels, as the latter inhibits uric acid excretion transporters. In phase 2 studies, dotinurad dose-dependently lowered serum uric acid levels and did not cause safety problems [NCT#02416167]. Furthermore, the serum uric acid lowering-effect of dotinurad was verified to be non-inferior to benzbromarone in our previous study [NCT#03100318].

However, as described above, uricosuric drugs such as benzbromarone is less prescribed and febuxostat is widely prescribed in clinical practice. Therefore, we conducted an additional study to compare the efficacy and safety of dotinurad versus febuxostat.

## Methods

### Study design

This was a phase 3, multicenter, randomized, double-blind, active-controlled, parallel-group, dose titration study conducted at 29 medical institutions in Japan.

### Inclusion and exclusion criteria

The inclusion criteria included the following: outpatients aged 20 years and older at the time of informed consent; the serum uric acid level on the first day of the run-in period ≥ 7.0 mg/dL (patients with a history of gout or with gouty tophi), ≥ 8.0 mg/dL (patients with any of the following: hypertension, diabetes mellitus, or metabolic syndrome, under treatment or follow-up), or ≥ 9.0 mg/dL (patients without any of the above conditions under treatment or follow-up) in reference to the Japanese guideline [1]; and patients who were classified as the uric acid underexcretion type, combined type, or normal type during the run-in period.

The exclusion criteria included the following: patients with unresolved gouty arthritis in the 14 days before the day of assignment/registration; patients with secondary hyperuricemia; patients who are revealed a renal calculus or who had a urinary calculus associated with clinical symptoms such as hematuria and back pain at first day of the run-in period; and patients with serum alanine aminotransferase (AST) or aspartate aminotransferase (ALT) ≥ 100 IU/L, estimated glomerular filtration rate (eGFR) < 30 mL/min/1.73 m^2^ at first day of the run-in period.

Patients who were on serum uric acid lowering medication or other medication that could affect the efficacy evaluation or safety underwent at least 14 days of washout after informed consent.

### Treatment

**Fig. 1:**
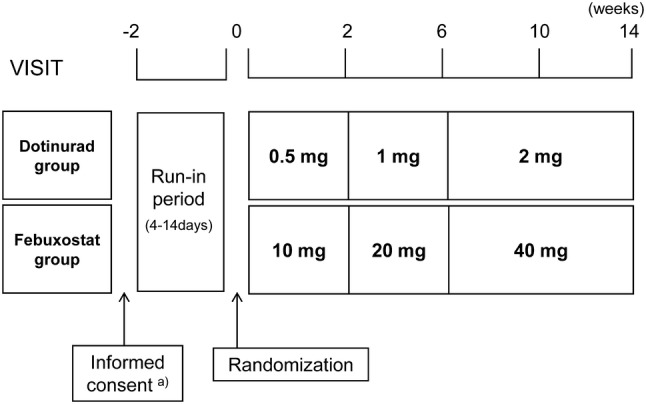
Study scheme. ^a)^Patients who were on prohibited concomitant medication underwent at least 14 days of washout after informed consent, before entering the run-in period

Patients were randomized to the dotinurad group or the febuxostat group at a 1:1 ratio on the final day of the run-in period. Dynamic allocation was employed with baseline serum uric acid levels and eGFR as factors for allocation. The study adopted dose titration (Fig. 1) to reduce the risk of gouty attacks due to a rapid fall in serum uric acid levels [2].

In order to minimize the risk of urinary calculus formation associated with increased urinary uric acid excretion, patients who met any of the following criteria concomitantly received urine alkalization drug (e.g., citrates): (1) a history of urinary calculus, (2) urinary pH < 6.0 at any time point after informed consent; and (3) necessity for drug determined by the investigator.

### Hyperuricemia classification

On the first day of the run-in period, a blood sample and a 60-min urine sample were collected and urinary extraction of uric acid (*E*_UA_ [mg/kg/h]) and uric acid clearance (C_UA_ [mL/min]) were determined to identify the patient’s hyperurisemic type according to the following criteria: (1) overproduction type, *E*_UA_ > 0.51 and *C*_UA_ ≥ 7.3; (2) underexcretion type, *E*_UA_ < 0.48 or *C*_UA_ < 7.3; (3) combined type, *E*_UA_ > 0.51 and *C*_UA_ < 7.3; and (4) normal type, 0.48 ≤ *E*_UA_ ≤ 0.51 and *C*_UA_ ≥ 7.3. Patients classified as the overproduction type were excluded from the study. The hyperuricemic type was determined in accordance with the then current Japanese guideline for the management of hyperuricemia and gout (second edition) [1] when the study was planned.

### Efficacy evaluations

The primary endpoint was the percent change in serum uric acid level from the baseline to the final visit. The secondary endpoints included the percentage of patients achieving a serum uric acid levels ≤ 6.0 mg/dL and serum uric acid levels, at selected time points.

### Safety evaluations

The investigator evaluated AEs and safety based on vital signs, 12-lead electrocardiography, laboratory tests, and physical examination. AEs were coded by the System Organ Class and Preferred Term (MedDRA version 21.0) and the causal relationship to the study drug and the severity and seriousness of each event were evaluated. An ADR was defined as an AE that was considered to be related to the study drug.

### Statistical analyses

The target number of patients was determined by the following method: With reference to the results of a phase 2b dotinurad study [NCT#02416167] and a phase 2b febuxostat study [16], the difference in the rate of serum uric acid lowering at the final visit between the two groups and its standard deviation (SD) were assumed to be 0% and 15%, respectively. The non-inferiority margin was set at 10% to show that dotinurad was not inferior more than one-third of the difference in the rate of serum uric acid lowering between febuxostat (39.81%) and placebo (0%). The sample size required to verify the non-inferiority of dotinurad to febuxostat with 90% power at a one-sided significance level of 2.5% was 100 patients per group, accommodating the comparison of safety results and the possibility of unused data due to dropouts.

Efficacy analyses were performed on the full analysis set (FAS) consisting of patients who received at least one dose of the study drug and had at least one efficacy measure evaluated after study treatment. The last observation carried forward method was employed to impute missing data on efficacy measures.

With regard to the primary endpoint, the summary statistics of the percent change in serum uric acid level from the baseline to the final visit were calculated for each group and the mean difference between the groups and its two-sided 95% CI were calculated to verify the non-inferiority of dotinurad to febuxostat. In addition, the summary statistics of the rate of serum uric acid lowering and its two-sided 95% CI were calculated for each subpopulation (based on baseline serum uric acid level and eGFR) in the FAS.

With regard to the secondary endpoints, the percentage of patients achieving a serum uric acid levels ≤ 6.0 mg/dL was determined for each group and then its two-sided 95% CI was calculated. The summary statistics of serum uric acid levels at selected time points and the two-sided 95% CI for the mean were calculated for each group.

Safety analyses were performed on the safety population (SP) consisting of patients who received at least one dose of the study drug and had evaluable safety information after study treatment. The number and proportion of patients with AEs and the number of AEs were calculated and tabulated. SAS software, version 9.3 (SAS Institute, Cary, NC, USA), was used in statistical analyses.

## Results

### Patient flowcharts and baseline characteristics

In the study, 406 patients were screened and 203 were excluded. Common reasons for exclusion were failure to meet the inclusion criteria and falling under any of the exclusion criteria. The remaining 203 were randomized to receive dotinurad (*n* = 102) or febuxostat (*n* = 101). Seven patients in each groups discontinued the study.

**Fig. 2:**
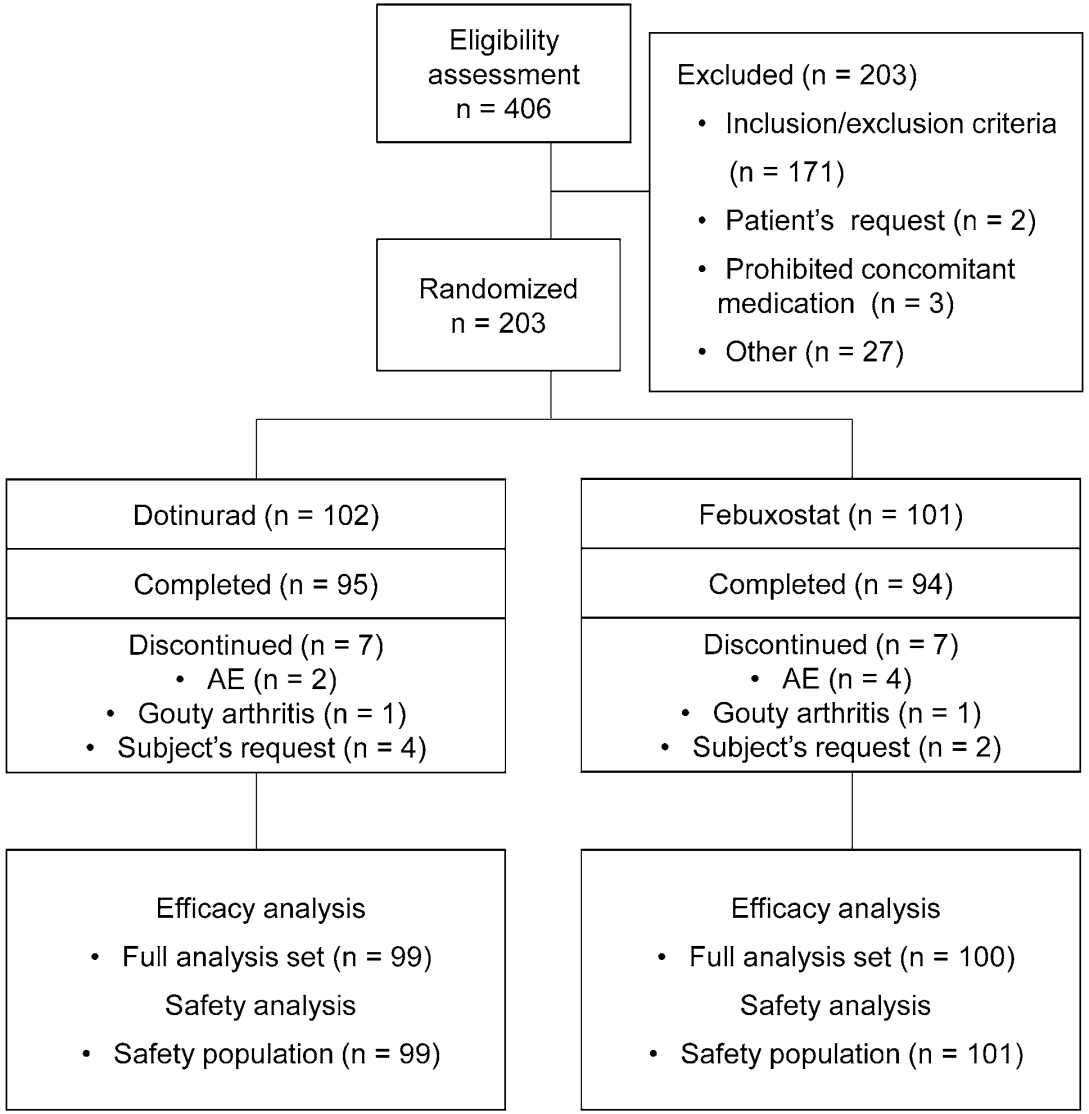
Flowchart of patients in this study

The FAS comprised 99 patients in the dotinurad group and 100 in the febuxostat group. The SP comprised 99 patients in the dotinurad group and 101 in the febuxostat group (Fig. 2). Reasons for exclusion from the SP (three patients in dotinurad group) were failure to receive any dose of the investigational products or absence of data for safety evaluation.

The demographic and other baseline characteristics in the FAS were similar in the two groups. The mean age was 56.1 years, 88.4% of patients were the underexcretion type, and 73.4% of patients had history of gouty arthritis. The mean serum uric acid level at baseline was 8.61 mg/dL in the dotinurad group and 8.67 mg/dL in the febuxostat group (Table 1).

**Table 1:** Summary of the baseline characteristics

Characteristics	Dotinurad (*n* = 99)	Febuxostat (*n* = 100)	Total (*n* = 199)
*Sex*			
No of patients (%)			
Male	99 (100.0)	100 (100.0)	199 (100.0)
Female	0 (0.0)	0 (0.0)	0 (0.0)
*Age (years)*			
Mean ± SD	55.1 ± 10.8	57.1 ± 10.6	56.1 ± 10.7
*Height (cm)*			
Mean ± SD	171.16 ± 5.66	169.73 ± 5.93	170.44 ± 5.83
*Body weight (kg)*			
Mean ± SD	78.63 ± 12.36	75.27 ± 12.37	76.94 ± 12.45
*BMI (kg/m*^2^*)*			
Mean ± SD	26.79 ± 3.68	26.08 ± 3.76	26.43 ± 3.73
No of patients (%)			
< 18.5 kg/m^2^	0 (0.0)	1 (1.0)	1 (0.5)
≥ 18.5 kg/m^2^ to < 22.0 kg/m^2^	10 (10.1)	10 (10.0)	20 (10.1)
≥ 22.0 kg/m^2^ to < 25.0 kg/m^2^	21 (21.2)	30 (30.0)	51 (25.6)
≥ 25.0 kg/m^2^	68 (68.7)	59 (59.0)	127 (63.8)
*Serum uric acid level (mg/dL)*			
Mean ± SD	8.61 ± 1.05	8.67 ± 1.06	8.64 ± 1.05
*eGFR (mL/min/1.73 m*^2^*)*^a^			
Mean ± SD	70.5 ± 16.2	69.7 ± 15.3	70.1 ± 15.7
*Treatment history*			
No of patients (%)			
No	15 (15.2)	16 (16.0)	31 (15.6)
Yes	84 (84.8)	84 (84.0)	168 (84.4)
*History of gouty arthritis*			
No of patients (%)			
No	25 (25.3)	28 (28.0)	53 (26.6)
Yes	74 (74.7)	72 (72.0)	146 (73.4)
*Gouty Tophi*			
No of patients (%)			
No	96 (97.0)	99 (99.0)	195 (98.0)
Yes	3 (3.0)	1 (1.0)	4 (2.0)
*Drinking habit*^b^			
No of patients (%)			
No	41 (41.4)	34 (34.0)	75 (37.7)
Yes	58 (58.6)	66 (66.0)	124 (62.3)
*Hyperuricemia classification*			
No of patients (%)			
Uric acid underexcretion type	87 (87.9)	89 (89.0)	176 (88.4)
Combined type/normal type	12 (12.1)	11 (11.0)	23 (11.6)

^a^eGFR (mL/min/1.73 m^2^) = 194 × Serum creatinine^−1.094^ × age^−0.287^

^b^Definition of drinking habit: consumption of alcohol on more than 3 days of the week and consumption of more than 500 mL of beer or 60 mL of whisky in a day

### Efficacy

#### The primary endpoint

The percent change in serum uric acid level from the baseline to the final visit (mean ± SD) was 41.82 ± 11.47% in the dotinurad group and 44.00 ± 10.63% in the febuxostat group. The mean difference in the rate of serum uric acid lowering between the dotinurad and febuxostat groups was − 2.17% (two-sided 95% CI − 5.26% to 0.92%) (Table 2). The lower limit of the two-sided 95% CI for the intergroup difference was above the prespecified non-inferiority margin (− 10%), demonstrating the non-inferiority of dotinurad to febuxostat.

**Table 2:** Results of primary efficacy endpoints

Group	Percent change in serum uric acid level from the baseline to the final visit (%)							
	No of patients	Mean ± SD	Two-sided 95% CI for the mean		Mean inter-group difference^a^ and two-sided 95% CI			One-sided *p* value^b^
			Lower limit	Upper limit	Inter-group difference^a^	Lower limit	Upper limit	
Dotinurad	99	41.82 ± 11.47	39.54	44.11	− 2.17	− 5.26	0.92	< 0.001
Febuxostat	100	44.00 ± 10.63	41.89	46.11				

^a^Dotinurad group − Febuxostat group

^b^Non-inferiority test of the dotinurad group versus the febuxostat group (non-inferiority margin = 10%)

#### The secondary endpoint

**Fig. 3:**
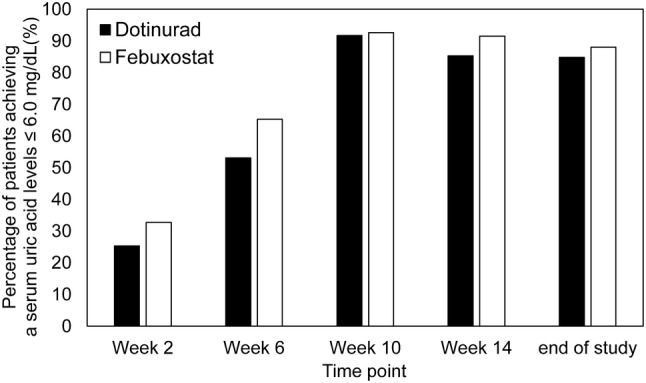
Percentage of patients achieveing a serum uric acid level ≤ 6.0 mg/dL at each time points

The percentage of patients achieving a serum uric acid levels ≤ 6.0 mg/dL gradually rose starting at Week 2. The achievement rate at the final visit was 84.8% in the dotinurad group and 88.0% in the febuxostat group, showing similarity between the two groups (Fig. 3).

**Fig. 4:**
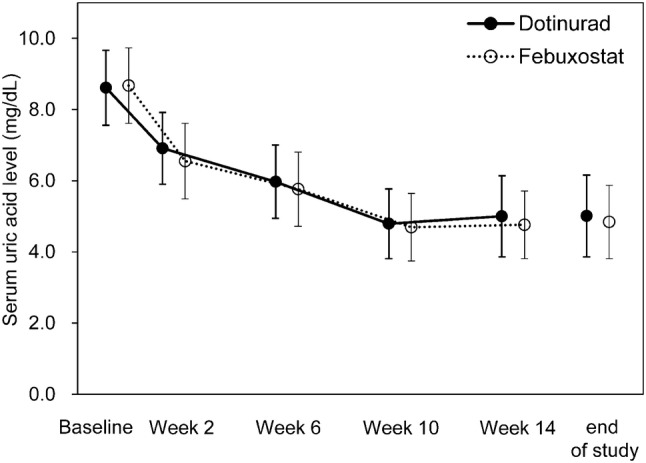
Time-course of the serum uric acid level. Error bars indicates standard deviation

The mean serum uric acid level gradually declined starting at Week 2 and kept at ≤ 6.0 mg/dL from Week 10 onward. The serum uric acid level (mean ± SD) at the final visit was 5.01 ± 1.15 mg/dL in the dotinurad group and 4.84 ± 1.03 mg/dL in the febuxostat group, showing similarity between the two groups (Fig. 4).

#### Subgroup analysis

No meaningful differences in the rate of serum uric acid lowering were noted between the baseline eGFR-based subgroups. Similarly, no meaningful differences were noted between the dotinurad and febuxostat groups within each subgroup (Table 3a, b).

**Table 3:** Subgroup analysis by category of eGFR at the baseline

(a) Percent change in serum uric acid level from baseline to final visit					
Category of eGFR^a^	Group	No of patients	Mean ± SD	Two-sided 95% CI for the mean	
				Lower limit	Upper limit
30 ≤ eGFR < 60	Dotinurad	22	41.36 ± 12.85	35.66	47.05
	Febuxostat	22	47.27 ± 10.52	42.60	51.93
60 ≤ eGFR < 90	Dotinurad	67	42.26 ± 10.91	39.60	44.92
	Febuxostat	68	43.70 ± 10.58	41.14	46.26
90 ≤ eGFR	Dotinurad	10	39.93 ± 12.99	30.64	49.22
	Febuxostat	10	38.82 ± 9.72	31.87	45.77

(b) Serum uric acid level at final visit and difference of serum uric acid level from baseline to final visit							
Category of eGFR^a^ group	No of patients	Serum uric acid level (mg/dL)			Difference of serum uric acid level (mg/dL)		
		Mean ± SD	Two-sided 95% CI for the mean		Mean ± SD	Two-sided 95% CI for the mean	
			Lower limit	Upper limit		Lower limit	Upper limit
30 ≤ eGFR < 60							
Dotinurad	22	5.10 ± 1.29	4.53	5.68	− 3.56 ± 1.12	− 4.06	− 3.07
Febuxostat	22	4.77 ± 1.01	4.32	5.22	− 4.31 ± 1.16	− 4.82	− 3.79
60 ≤ eGFR < 90							
Dotinurad	67	4.95 ± 1.10	4.68	5.22	− 3.64 ± 1.11	− 3.91	− 3.37
Febuxostat	68	4.84 ± 1.07	4.58	5.10	− 3.77 ± 1.05	− 4.02	− 3.51
90 ≤ eGFR							
Dotinurad	10	5.17 ± 1.20	4.31	6.03	− 3.46 ± 1.30	− 4.39	− 2.53
Febuxostat	10	5.00 ± 0.78	4.44	5.56	− 3.20 ± 0.93	− 3.87	− 2.53

^a^eGFR (mL/min/1.73 m^2^) = 194 × Serum creatinine^−1.094^ × age^−0.287^

### Safety

The incidence of AEs in the dotinurad and febuxostat groups was 52.5% and 51.5%, respectively. The incidence of ADRs in the dotinurad and febuxostat groups was 17.2% and 19.8%, respectively (Table 4). The AEs were judged mostly mild or moderate in severity by the investigator.

**Table 4:** Summary of adverse events

	Dotinurad			Febuxostat		
	(*n* = 99)			(*n* = 101)		
	No of patients	Incidence (%)	No of events	No of patients	Incidence (%)	No of events
AEs	52	(52.5)	89	52	(51.5)	105
ADRs	17	(17.2)	21	20	(19.8)	37
SAEs excluding deaths	3	(3.0)	3	2	(2.0)	2
AEs leading to treatment discontinuation	2	(2.0)	2	5	(5.0)	5
AEs (PT) with incidence ≥ 5%						
Nasopharyngitis	10	(10.1)	12	4	(4.0)	5
Gouty arthritis	3	(3.0)	4	6	(5.9)	8
Upper respiratory tract inflammation	2	(2.0)	2	6	(5.9)	6

*AE* adverse event, *ADR* adverse drug reaction, *SAE*serious adverse event, *PT* preferred term

The incidence of serious adverse events (SAEs) in the dotinurad and febuxostat groups was 3.0% and 2.0%, respectively. SAEs included colon cancer, neoplasm malignant, and gastric cancer (one patient each) in the dotinurad group and liver disorder and adenocarcinoma of colon (one patient each) in the febuxostat group. Among these, only liver disorder was judged to be an ADR by the investigator.

The incidence of gouty arthritis in the dotinurad and febuxostat groups was 3.0% and 5.9%, respectively: one patient at 1 mg and three patients at 2 mg (one of whom experienced gouty arthritis at 1 mg as well) in the dotinurad group and three patients each at 20 and 40 mg in the febuxostat group. The investigator determined that all events of gouty arthritis were mild or moderate in severity. No urinary calculus was found in the patients of either group.

## Discussion

In this study, the serum uric acid lowering effect of dotinurad showed to be non-inferior to that of febuxostat. The great majority of dotinurad-treated patients achieved a serum uric acid level ≤ 6.0 mg/dL, the treatment goal in Japan, after a dose increase to 2 mg. This efficacy was maintained until the final visit, as seen in febuxostat-treated patients. The serum uric acid lowering effect of febuxostat observed in this study was comparable to that in other clinical studies.

Dotinurad-treated subjects had a lower rate of serum uric acid lowering compared to febuxostat-treated ones at individual time points, despite comparability in the mean rate of serum uric acid lowering in the two treatment groups. This could partially be explained by a slightly higher proportion of subjects with body mass index (BMI) ≥ 25.0 kg/m^2^ in the dotinurad group (68.7%) than in the febuxostat group (59.0%) (Table 1). Some researchers have reported that greater BMI affects the therapeutic efficacy of uric acid lowering medications [17].

The results of subgroup analysis demonstrated no effect of renal function on the rate of serum uric acid lowering. In clinical studies of patients with renal dysfunction (30 mL/min/1.73 m^2^ ≤ eGFR < 60 mL/min/1.73 m^2^), dotinurad had no notable impact on pharmacokinetics and pharmacodynamics [NCT# 02347046]. Therefore, it is presumed that dotinurad can be used for treatment without dose adjustment in patients with mild or moderate renal dysfunction.

The incidences of AEs or ADRs did not differ notably between the groups and most AEs were mild or moderate in severity. The incidence of gouty arthritis was slightly lower in the dotinurad group than in the febuxostat group, without any differences of special note. In this study, no urinary calculus was found in patients in either group.

The incidence of SAEs in the dotinurad and febuxostat groups was 3.0% and 2.0%, respectively. Among these, only liver disorder in the febuxostat group was considered to be an ADR. This patient had AST and ALT abnormalities at Weeks 2 (AST of 70 U/L and ALT of 55 U/L at the baseline; AST of 1110 U/L and ALT of 612 U/L at Weeks 2). These elevations were transient and a trend toward normal values was observed before treatment discontinuation.

Liver disorders were often reported as an ADR of antihyperuricemic. For instance, benzbromarone is reported to be associated with fulminant hepatitis and jaundice [18]. Therefore, benzbromarone is used only in some countries, and is contraindicated in patients with hepatic impairment in Japan. Post-marketing surveillance of febuxostat has revealed that it rarely causes hepatic failure reported as serious ADRs [19]. In this study, liver function related ADRs occurred only the febuxostat group and comprised ‘gamma-glutamyltransferase (γ-GTP) increased’ (one patient), ‘liver function test abnormal’ (two patients), and ‘liver disorder’ (one patient). The ‘liver disorder’ was severe; others were mild. Table 5 shows an excerpt of laboratory liver function tests (AST, ALT, and *γ*-GTP). The mean values of these test items remained stable in both groups from baseline through the final visit. AST levels were within the reference range at baseline and then exceeded the upper limit of the reference range after study treatment in six dotinurad-treated subjects and 12 febuxostat-treated subjects. In summary, dotinurad therapy did not result in any signs of liver disorders, which are safety concerns associated with the existing drugs.

**Table 5:** Summary of laboratory data (AST, ALT, *γ*-GTP, Creatinine)

	Dotinurad (*n* = 99)	Febuxostat (*n* = 101)
	Mean ± SD	Mean ± SD
AST (U/L)		
Run-in period	26.0 ± 9.5	28.1 ± 11.3
Final visit	24.0 ± 8.5	27.8 ± 10.7
ALT (U/L)		
Run-in period	30.7 ± 16.9	30.1 ± 17.5
Final visit	28.0 ± 16.8	30.1 ± 17.7
γ-GTP (U/L)		
Run-in period	66.5 ± 54.8	67.3 ± 53.4
Final visit	66.4 ± 69.1	65.9 ± 58.0
Creatinine (mg/dL)		
Run-in period	0.924 ± 0.179	0.922 ± 0.172
Final visit	0.939 ± 0.201	0.931 ± 0.196

Recently approved in the US and Europe, lesinurad is the same SURI as dotinurad and is prescribed with an XOI. It is reported that high-dose lesinurad may increase serum creatinine levels and cause serious ADRs, e.g. acute renal failure [20]. In the present study, one febuxostat-treated patient experienced renal impairment as an ADR related to renal function, which was mild in severity. Table 5 shows serum creatinine level. No conspicuous changes in the mean creatinine level from baseline were noted in either group. Similarly, there were no significant changes in any other renal function test items (urinary *N*-acetyl-*β*-d-glucosaminidase [NAG], urinary *α*1-microglobulin [AMG], and urinary *β*2-microglobulin [BMG]) from baseline. In the febuxostat group, NAG increased (two subjects), AMG increased (one subject), and BMG increased (four subjects) were reported as ADRs, whereas in the dotinurad group, only BMG increased (one subject) was reported. All of these ADRs were mild in severity. In summary, dotinurad therapy did not show any signs related to renal failure or renal disorders which are safety concerns reported with lesinurad.As mentioned above, in Japan, most hyperuricemic patients are of the underexcretion type. In addition, it has been reported that drug selection based on pathological conditions resulted in better control of serum uric acid level [21]. Considering the above the role in clinical practice of uric acid lowering agents that work by stimulation of uric acid excretion will likely become important.

The results of the study demonstrated the non-inferiority of dotinurad to febuxostat in terms of efficacy and a favorable safety profile in hyperuricemic patients, excluding those of the uric acid overproduction type. Dotinurad is a promising medication that may provide optimal treatment to numerous hyperuricemic patients.
